# Procyanidin displayed a synergistic effect with roxadustat on renal anemia in mice

**DOI:** 10.3389/fphar.2025.1678846

**Published:** 2025-11-12

**Authors:** Mingyang Cui, Zhe Wang, Jiaxin Shi, Wenbin Wu, Jin Tao, Yujing Han, Zihui Yang, Junjie Luo, Yongting Luo, Peng An, Mingyue Sun

**Affiliations:** 1 Department of Nutrition and Health, China Agricultural University, Beijing, China; 2 Beijing National Day Wonder School, Beijing, China; 3 Beijing No.35 High School, Beijing, China; 4 Beijing No.8 High School, Beijing, China; 5 Xiyuan Hospital, China Academy of Chinese Medical Sciences, Beijing, China

**Keywords:** renal anemia, kidney injury, procyanidin, roxadustat, iron metabolism

## Abstract

**Introduction:**

Chronic kidney disease (CKD) often leads to renal anemia, a complication traditionally treated with erythropoiesis-stimulating agents (ESAs) and iron supplementation. However, these treatments may increase cardiovascular risks. Procyanidin, a compound found in black goji berries and mulberries, has known kidney-protective properties. This study aimed to explore the synergistic effects of procyanidin and roxadustat in alleviating renal anemia in CKD.

**Methods:**

A mouse model of CKD was induced by administering adenine, followed by treatment with procyanidin (250 mg/kg/day), roxadustat (5 mg/kg/day), or both. The effects on erythropoiesis, kidney function, iron metabolism, and inflammation were assessed through hematological analyses, histopathology, and gene expression profiling.

**Results:**

Procyanidin alone improved erythropoiesis, kidney function, and reduced renal injury. Roxadustat enhanced erythropoiesis and iron metabolism but showed no significant effect on kidney function. The combination of both compounds resulted in superior improvements in hematological parameters, reduced kidney injury, fibrosis, and inflammation, and enhanced iron mobilization and absorption. Notably, procyanidin mitigated the thrombopoiesis induced by roxadustat.

**Discussion:**

The combination of procyanidin and roxadustat provides a promising therapeutic approach for renal anemia by improving erythropoiesis, kidney function, and iron metabolism while reducing adverse effects, such as thrombosis. These findings suggest that procyanidin may enhance the safety and efficacy of roxadustat in CKD-related renal anemia. Further studies are needed to optimize treatment regimens for clinical application.

## Introduction

Chronic kidney disease (CKD) is a growing global public health concern, with rising incidence and mortality rates since 1990. CKD is a heterogeneous condition caused by various underlying diseases, such as diabetes and hypertension (Webster et al., 2017). In 2017, the global prevalence of CKD reached 9.1%, with approximately 1.2 million related deaths (GBD Chronic Kidney Disease Collaboration, 2020).

Renal anemia, one of the most common complications of CKD, imposes a significant burden on patients and healthcare systems (St Peter et al., 2018; Mikhail et al., 2017). Closely linked to the poor prognosis of CKD, its prevalence substantially increases with disease progression (KDOQI National Kidney Foundation, 2006). Typically characterized by normal red blood cell morphology and pigmentation but impaired proliferation, renal anemia becomes more prevalent as the glomerular filtration rate (GFR) declines (Babitt and Lin, 2012). The mechanism of renal anemia is complex, involving shortened red blood cell lifespan, deficiency and dysfunction of erythropoietin (EPO), iron metabolism disorders, and other factors (Podkowińska and Formanowicz, 2020). Oxidative stress activates calcium channels on erythrocyte membranes, promoting calcium influx and potassium channel activation, leading to cell contraction and apoptosis (Bissinger et al., 2019). Meanwhile, it damages membrane proteins like ankyrin and spectrin, reducing red blood cell lifespan from 120 days to 80 days (Drai et al., 2001). In CKD patients, hepcidin levels rise due to reduced renal clearance and increased inflammatory factors. Hepcidin binds to ferroportin, promoting its degradation and inhibiting intestinal iron absorption and macrophage iron release, resulting in functional iron deficiency (Ganz and Nemeth, 2016; Nemeth et al., 2004). Additionally, the chronic inflammatory state in CKD patients can affect the glycosylation modification of transferrin (TF), leading to changes in the proportion of TF variants, which in turn impairs iron transport efficiency and exacerbates anemia (Formanowicz and Formanowicz, 2012). Although intravenous iron supplementation corrects iron deficiency, free iron ions promote reactive oxygen species (ROS) production via the Fenton reaction, exacerbating oxidative stress (Podkowińska and Formanowicz, 2020). Renal anemia significantly impacts CKD patients, causing severe quality of life impairment, increased cardiovascular disease risk, cognitive dysfunction, and elevated mortality (Hanna et al., 2021). Surveys show that 30%–50% of end-stage kidney disease patients exhibit an inflammatory state with markedly elevated C-reactive protein and proinflammatory cytokines (IL-1, IL-6, TNF-α) (Stenvinkel and Alvestrand, 2002; Owen and Lowrie, 1998; Stenvinkel et al., 2002; Yeun et al., 2000; Kimmel et al., 1998). Persistent inflammation in CKD is strongly associated with oxidative stress, infection, impaired cytokine clearance, and dialysis (Stenvinkel and Alvestrand, 2002). Within the context of systemic inflammation, inflammatory factors further contribute to the risk of renal anemia (Wojtaszek et al., 2021).

EPO plays a crucial role in stimulating erythropoiesis in the bone marrow. It also drives hemoglobin synthesis via increasing iron absorption and mobilization in the body. Insufficient EPO production in the kidneys is the primary cause of renal anemia. CKD and deficient EPO will disturb iron metabolism, and further exacerbate the erythropoiesis and the symptoms of renal anemia. The human body contains about four to five grams of iron, and most iron existed in hemoglobin and the reticuloendothelial system (e.g., macrophages), while a smaller portion of iron is stored in the liver and spleen (Ogawa et al., 2018). When the body requires iron (e.g., iron deficiency, hypoxia, and blood loss etc.), it will reduce the synthesis of iron-regulatory hormone hepcidin in the liver. Then more iron will be released from iron stores (e.g., macrophages and liver) and absorbed from small intestine into circulation (Babitt and Lin, 2010).

The traditional clinical treatments for renal anemia include administrations of erythropoiesis-stimulating agents (ESAs) and iron supplements. The latest research has shown that anti-bone morphogenetic protein 6 (anti-BMP-6) antibodies exert favorable therapeutic effects in rodent models of renal anemia, though their clinical application remains in development (Badura et al., 2024; Petzer et al., 2020). In addition, drugs targeting hypoxia-inducible factor (HIF)-prolyl hydroxylase inhibitor (PHI) axis provide a new therapeutic strategy for renal anemia. Roxadustat is the first HIF-PHI drug approved for the treatment of renal anemia (Dhillon, 2019). Under normal physiological conditions, HIF is ubiquitinated and degraded in proteasomes through the catalysis of prolyl hydroxylases (Bruick and McKnight, 2001). Roxadustat prevents HIF degradation via inhibiting prolyl hydroxylases, thereby promoting HIF expression and subsequently upregulating the expression of HIF downstream genes, such as EPO. Roxadustat can also partially improve the poor responsiveness of ESAs by modulating gut microbiota (Coll et al., 2024). Compared with ESAs, roxadustat offers some advantages, such as the convenience of administration, avoidance of infection risks associated with injections, and improvement of iron metabolism (Dhillon, 2019; Li et al., 2020). However, the administration of roxadustat is associated with increased risks of cardiovascular diseases, such as hypertension and thromboembolism (St Peter et al., 2018; Barratt et al., 2021; Fishbane et al., 2021; Chung et al., 2023). Therefore, safer and more effective strategies are needed to reduce the related adverse effects of roxadustat and provide better therapeutic outcomes.

Procyanidin, a compound found in black goji berries, mulberries, and hawthorns, has anti-inflammatory, antioxidant, and renal protective effects (Liu et al., 2020; Zhao et al., 2022). Widely used in traditional Chinese medicine (Sun et al., 2022), it may offer a safer alternative or complementary therapy. Therefore, this study aims to investigate whether procyanidin can improve renal anemia, and whether it has a synergistic effect when combined with roxadustat.

## Materials and methods

### Animals and treatments

All animal experimental operations received approval, and were conducted according to the guidelines of the Animal Welfare and Ethics Review Committee of China Agricultural University (No. AW71803202-5-1). Male C57BL/6J mice were housed in a specific pathogen-free animal facility of China Agricultural University. The temperature in the animal facility was maintained at 24 °C ± 2 °C, with a humidity of 55% ± 5%. Mice were placed in individually ventilated, pathogen-free cages. Five mice in each group, a total of 25 mice. Mice were kept in an environment with a 12-h light/dark cycle with supply of standard rodent diet and water. Mice were randomly divided into five groups: control group, renal anemia group (RA group), procyanidin intervention group (Pro group), Roxadustat intervention group (Rox group), and a group receiving both procyanidin and roxadustat intervention (RA + Pro + Rox group). After a week of acclimatization, mice in the RA, Pro, Rox, and RA + Pro + Rox groups received an administration of 50 mg/kg/d adenine (Sigma-Aldrich, A8626, United States) for 5 weeks via oral gavage, while mice in the control group received an equal volume of 0.9% saline. Procyanidin or/and roxadustat interventions were initiated at the second week, the onset of renal anemia symptoms according to a previous study (Wang et al., 2018). Two weeks after the start of adenine administration, mice in the Pro group received 250 mg/kg/d procyanidin (Sigma-Aldrich, PHL89552, United States, purity >95%) via oral gavage and 0.9% saline via intraperitoneal injection for 3 weeks. Mice in the Rox group received 0.9% saline via oral gavage and 5 mg/kg/d roxadustat (MedChemExpress, HY-13426, United States) via intraperitoneal injection for 3 weeks. Mice in the RA + Pro + Rox group received both 250 mg/kg/d procyanidin via oral gavage and 5 mg/kg/d roxadustat via intraperitoneal injection for 3 weeks. After 2 weeks, the Control and RA groups received both 0.9% saline gavage and intraperitoneal injection. All mice in the experimental group were given adenine for 5 weeks to induce renal anemia. After intervention, mice were weighed and sacrificed to collect the whole blood, kidneys, livers, small intestine and other tissues.

### Hematological and serological measurements

After anesthesia with isoflurane (RWD Life Science, R510-22-10, China), whole blood was collected from the eyeballs of mice, and dispensed into anticoagulation tubes containing EDTA-K2 and coagulation-promoting tubes with separation gel, respectively. The anticoagulation tubes were used for hematological parameters measuring using an automated hematology analyzer (Nihon kohden MEK-7300P, Japan). The blood collected in the coagulation-promoting tubes was left at room temperature for 3 h, then centrifuged to obtain serum. The creatinine and urea content were measured using commercial kits (Solarbio, BC4915, China; and Solarbio, BC1535, China) according to the manufacturer’s instructions.

### Histopathology

The left kidney of the mouse underwent dehydration through a gradient of ethanol, and subsequent immersion in xylene. Then tissue was immediately followed by gradual wax immersion through three levels of wax solution. The kidney was then embedded in a paraffin embedding machine and frozen for solidification. The resulting wax block was sliced into sections. Renal tissue sections were subsequently stained according to the instructions of a commercial hematoxylin and eosin (H&E) staining kit (G1120, Solarbio, China), periodic acid-Schiff (PAS) staining kit (G1281, Solarbio, China), and Masson’s trichrome staining kit (G1340, Solarbio, China).

### Real-time quantitative PCR (qPCR)

According to the manufacturer’s protocol, total RNA was extracted from kidney, liver, and duodenum using Trizol, followed by reverse transcription using a reverse transcription kit according to the manufacturer’s instructions (11141S60, Yeasen, China). The cDNA obtained from reverse transcription was aliquoted and used for qPCR analysis. *β-Actin* was used as the internal reference gene. The primer sequences for genes detected using qPCR are presented in Supplementary Table S1. Results were quantitatively analyzed by the 2^−ΔΔCT^ method.

### Tissue non-heme iron measurement

Tissue non-heme iron concentrations were determined by reacting non-heme iron with a bathophenanthroline reagent and then adoped the classical colorimetric method. The tissue is completely digested with an acid digesting solution (3 M hydrochloric acid with 0.61 M trichloroacetic acid). The tissue iron working solution was prepared by mixing 1.86 mM bathophenanthroline sulfonate, saturated sodium acetate, and ddH_2_O in a ratio of 1:5:5. Subsequently, 200 μL of the tissue iron working solution was added to a 96-well plate. After centrifuging the digesting solution, 10 μL of the sample supernatant was taken and added to the tissue iron working solution. Meanwhile, 10 μL of iron standard solution is transferred into the tissue iron working solution as the iron standard, and 10 μL of acid digesting solution is used as the blank control. After thoroughly mixied the solutions in the 96-well plate, allowed them to develop color for 10 min, and measured the absorbance at 535 nm using a microplate spectrophotometer (Xu et al., 2024). The concentration of tissue non-heme iron is expressed as micrograms of iron per gram wet weight of tissue (μg/g wet weight).

### Statistical analysis

All experimental data were analyzed using the GraphPad Prism 9.0 (GraphPad Software, San Diego, CA, United States) and expressed as mean ± standard deviation. One-way analysis of variance (ANOVA) and Tukey *post hoc* test were used for multiple comparisons (group size ≥3). *P* values were two-sided, and *P* < 0.05 was considered as statistically significant. Significant differences between groups were indicated by *, where * indicates *P* < 0.05; ** indicates *P* < 0.01; *** indicates *P* < 0.001; n. s. Indicates no significant difference.

## Results

### Procyanidin displayed synergistically improving effect with roxadustat on erythropoiesis

**FIGURE 1:**
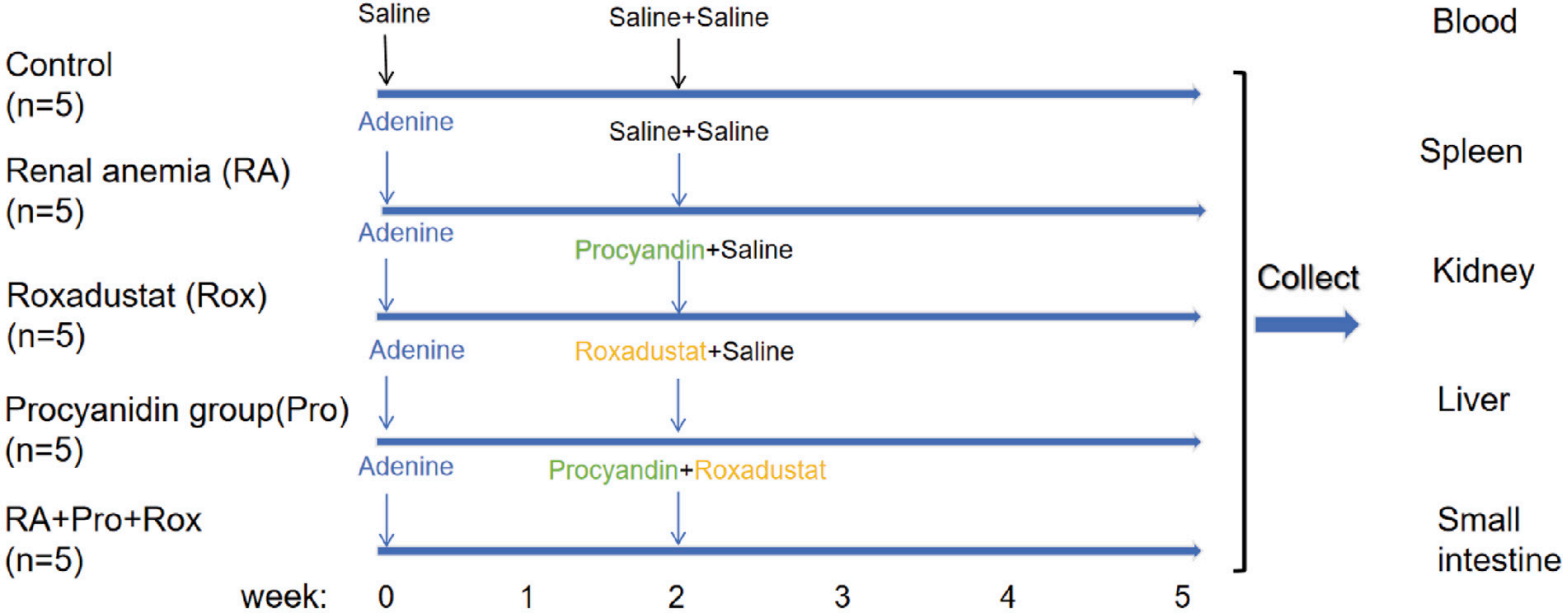
A summary of the process of animal experiment.

**FIGURE 2:**
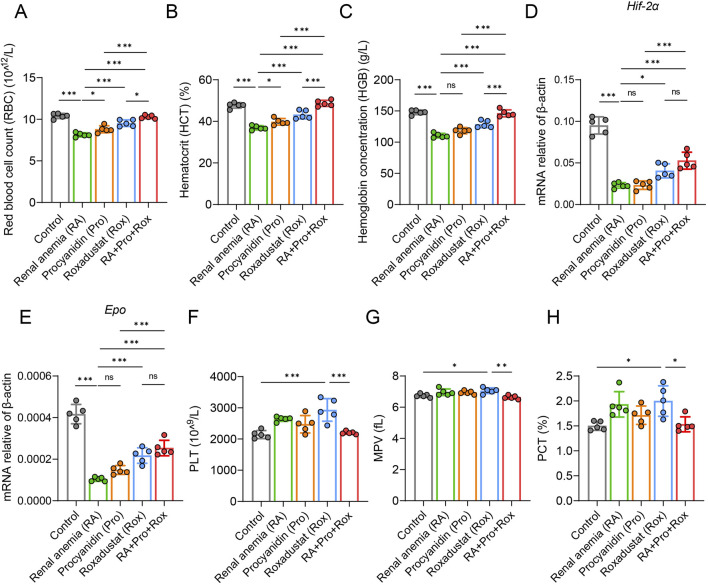
Procyanidin displayed synergistically improving effect with roxadustat on erythropoiesis without promoting thrombopoiesis. **(A)** Red blood cell count (RBC), **(B)** hematocrit (HCT), and **(C)** hemoglobin concentration (HGB) were measured using anticoagulant whole blood. Transcription level of **(D)** *Hif-2α* and **(E)** *Epo* in renal tissue. **(F)** platelet count (PLT), **(G)** mean platelet volume (MPV), and **(H)** plateletcrit (PCT) were measured using anticoagulant whole blood. Data were expressed as the mean ± standard deviation. One-way analysis of variance (ANOVA) and Tukey *post hoc* test were used to compare the group differences. Significant differences between groups were indicated by *, where * indicates *P* < 0.05; ** indicates *P* < 0.01; *** indicates *P* < 0.001; ns indicates no significant difference.

The process of animal experiment was summarized in Figure 1. To evaluate whether anemia symptoms were present in the renal anemia (RA) group of mice, and the efficacy of three intervention groups in ameliorating anemia, hematological parameters were analyzed. Key indicators for anemia include red blood cell count (RBC), hematocrit (HCT), and hemoglobin concentration (HGB) were shown in Figures 2A–C. RA group exhibited significantly decreased RBC, HCT, and HGB levels when compared with the control group (*P* < 0.001). For the Rox group (treated with roxadustat), RBC, HCT, and HGB levels were significantly elevated compared with the RA group (P < 0.001). Notably, RA + Pro + Rox group (treated with both procyanidin and roxadustat) displayed larger increase of RBC, HCT, and HGB levels compared with Rox group (Figures 2A–C). These findings indicate that RA mice receiving procyanidin and roxadustat (RA + Pro + Rox group) displayed a superior effect on erythropoiesis than those receiving procyanidin or roxadustat alone.

Subsequently, qPCR analysis of HIF and its downstream target EPO revealed significantly reduced *Hif-2α* and *Epo* expressions in the RA group when compared with the control group (Figures 2D,E). Procyanidin intervention (Pro group) seems to have no influence on *Hif-2α* and *Epo* expressions. In contrast, RA mice receiving roxadustat alone (Rox group) or procyanidin and roxadustat (RA + Pro + Rox group) significantly elevated *Hif-2α* and *Epo* expression levels (Figures 2D,E).

These results suggest procyanidin displayed synergistic effect with roxadustat on erythropoiesis without further upregulating *Hif-2α* and *Epo*.

### Procyanidin reduced thrombopoiesis induced by roxadustat

Roxadustat stimulates erythropoiesis by enhancing the release of EPO, which concurrently promotes the production of both red blood cells and platelets. However, excessive platelet generation may lead to abnormal coagulation, predisposing to thrombosis within vessels. Long-term roxadustat use has been documented to induce cardiovascular complications such as hypertension and thrombosis (Chen et al., 2019; Vaziri, 2008).

Monitoring platelet levels serves as an primary assessment of coagulation function alterations, including platelet count (PLT), mean platelet volume (MPV), and plateletcrit (PCT) (Figures 2F–H). Our study indicate that roxadustat treatment resulted in significant elevations in platelet count (PLT), mean platelet volume (MPV), and plateletcrit (PCT) compared with control group (*P* < 0.05). RA mice in the Pro group (treated with procyanidin) or RA + Pro + Rox group (treated with both procyanidin and roxadustat) exhibited significant reductions in these platelet indices compared with those in the Rox group (treated with roxadustat alone). These results suggest that concomitant administration of procyanidin with roxadustat mitigates thrombopoiesis induced by roxadustat.

### Procyanidin and roxadustat synergistically improved kidney function and alleviated kidney injury

**FIGURE 3:**
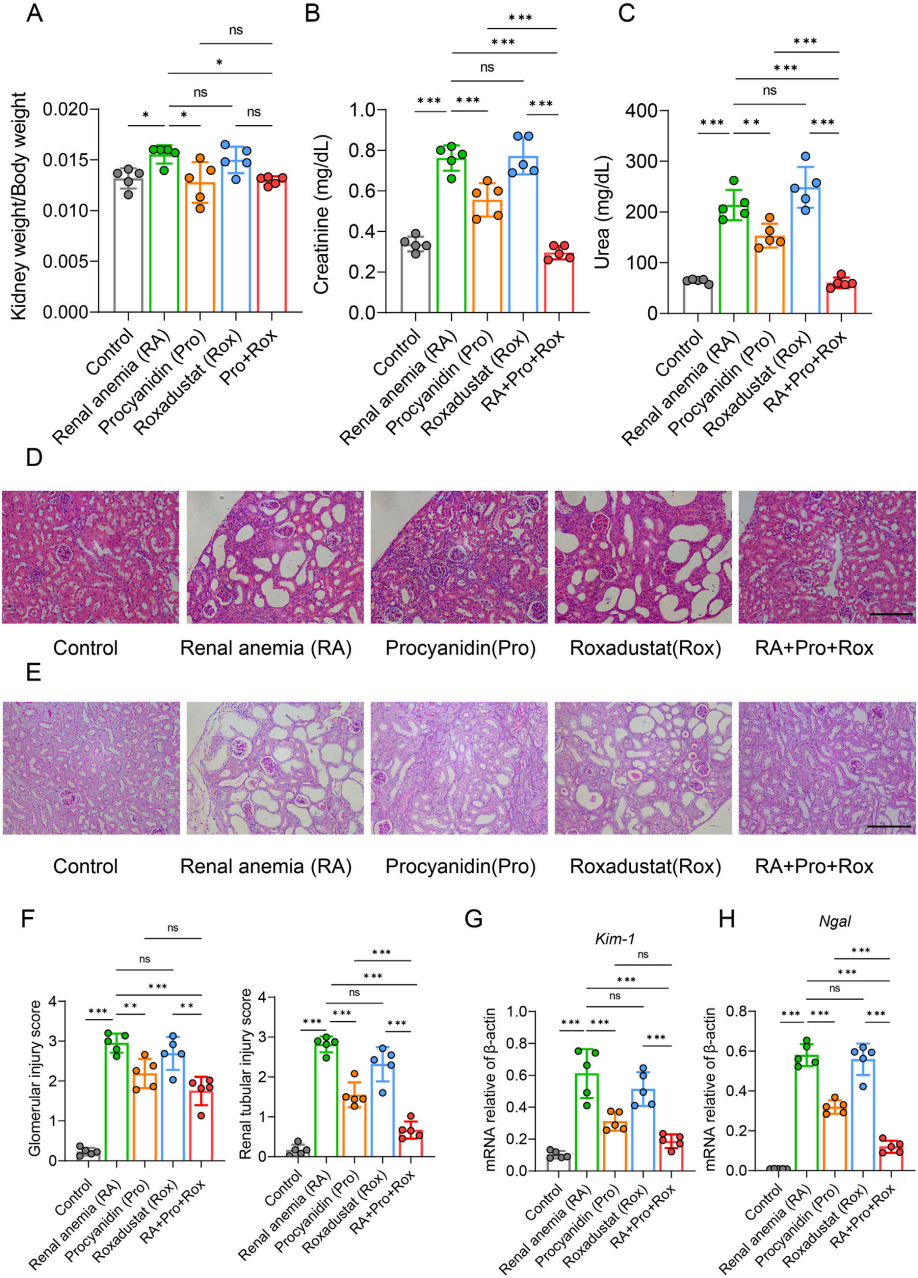
Procyanidin and roxadustat synergistically improved kidney function and alleviated kidney injury. **(A)** Comparison of renal coefficient in mice. Renal function indicators **(B)** serum creatinine and **(C)** urea concentrations were measured. **(D)** Results of hematoxylin and eosin (H&E) staining of kidney. **(E)** Periodic acid-Schiff (PAS) staining. **(F)** Histopathological scoring of kidney tissues. Kidney injury markers **(G)** *Kim-1* and **(H)** *Ngal* mRNA expressions were measured in the kidney. Data were expressed as the mean ± standard deviation, scale = 100 μm. One-way analysis of variance (ANOVA) and Tukey *post hoc* test were used to compare the group differences. Significant differences between groups were indicated by *, where * indicates *P* < 0.05; ** indicates *P* < 0.01; *** indicates *P* < 0.001; ns indicates no significant difference.

Observation of kidney coefficient (kidney weight/body weight) revealed compensatory hyperplasia in RA mice group, which was mitigated by either procyanidin treatment alone (Pro group) or combined intervention (RA + Pro + Rox group) (Figure 3A).

Serum creatinine and urea are two major indicators of renal filtration function in mice (Figures 3B,C). Serum creatinine and urea levels were significantly reduced in RA mice receiving procyanidin (Pro group), and those receiving procyanidin and roxadustat (RA + Pro + Rox group). In contrast, RA mice receiving roxadustat alone (Rox group) displayed unchanged serum creatinine and urea levels.

Consistent findings were observed from H&E staining and pathological scoring (Figure 3D). In RA mice group, H&E staining of kidney revealed glomerular atrophy, dilation of renal capsular cavities, dilation of renal tubular lumens, thinning of tubular walls, and the presence of vacuolation. Improvements in renal pathology were observed in RA mice receiving procyanidin (Pro group), and those receiveing procyanidin and roxadustat (RA + Pro + Rox group). In contrast, RA mice receiveing roxadustat alone (Rox group) exhibited a similar kidney pathology with the RA mice. Further assessment of glomerular and tubular lesions using PAS staining (Figure 3E) and semi-quantitative scoring (Figure 3F) yielded consistent results with H&E staining, indicating that both procyanidin treatment alone and combined intervention with roxadustat improved renal pathology.

Key markers of tubular injury include Kidney injury molecule 1 (KIM-1) and Neutrophil gelatinase-associated lipocalin (NGAL) were measured. KIM-1, a transmembrane glycoprotein expressed in proximal tubular epithelial cells, is minimally expressed in the healthy liver, kidney, and spleen, but significantly upregulated in regenerating proximal tubular epithelial cells after injury. Similarly, NGAL is upregulated and released upon renal injury. Examination of renal injury markers in mouse kidney tissues showed that RA mice receiving procyanidin alone (Pro group) or those receiving procyanidin and roxadustat (Pro + Rox group) significantly improved renal injury, whereas RA mice receiving roxadustat alone (Rox group) had no effect on renal injury (Figures 3G,H). Notably, RA mice receiving both procyanidin and roxadustat (RA + Pro + Rox group) yielded the most pronounced improvement in renal injury.

### Procyanidin and roxadustat synergistically improved renal fibrosis

**FIGURE 4:**
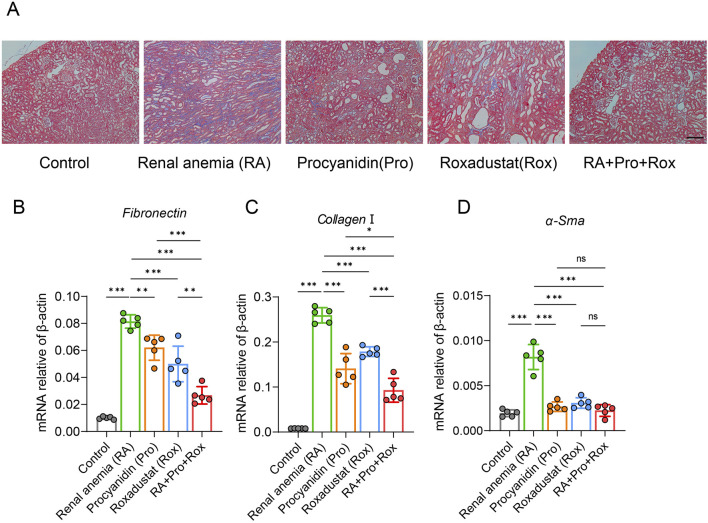
Procyanidin and roxadustat synergistically improved renal fibrosis. **(A)** Masson’s trichrome staining of kidney tissues. **(B–D)** Transcriptional expression levels of renal fibrosis markers: **(B)** *Fn,* **(C)** *Collagen I,* and **(D)** *α-Sma*. Data are expressed as the mean ± standard deviation, scale = 100 μm. One-way analysis of variance (ANOVA) and Tukey *post hoc* test were used to compare the group differences. Significant differences between groups were indicated by *, where * indicates *P* < 0.05; ** indicates *P* < 0.01; *** indicates *P* < 0.001; ns indicates no significant difference.

Masson’s trichrome staining revealed obvious renal tubulointerstitial fibrosis (blue collagen area) in RA mice (Figure 4A). Mice receiving procyanidin (Pro group), roxadustat (Rox group), or both (RA + Pro + Rox group) exhibited varying degrees of attenuation in fibrosis levels (Figure 4A). This indicates that both single and combined interventions can effectively alleviate tubulointerstitial fibrosis. Following severe renal tissue damage, fibroblast activation leads to enhanced contractility and increased secretion of inflammatory mediators, resulting in excessive accumulation of extracellular matrix components such as α-smooth muscle actin (α-SMA), collagen I, and fibronectin (FN), facilitating differentiation of fibroblasts into myofibroblasts and ultimately structural destruction. RA mice displayed significant upregulation of fibrotic markers compared with control mice, including *α-Sma, Collagen I,* and *Fn*, whose expressions significantly decreased in mice treated with procyanidin, roxadustat or both (Figures 4B–D). RA + Pro + Rox group exhibited the most pronounced reduction in fibrosis, indicating combined intervention yielded superior improving effect on renal fibrosis.

### Procyanidin and roxadustat synergistically reduced kidney inflammation

**FIGURE 5:**
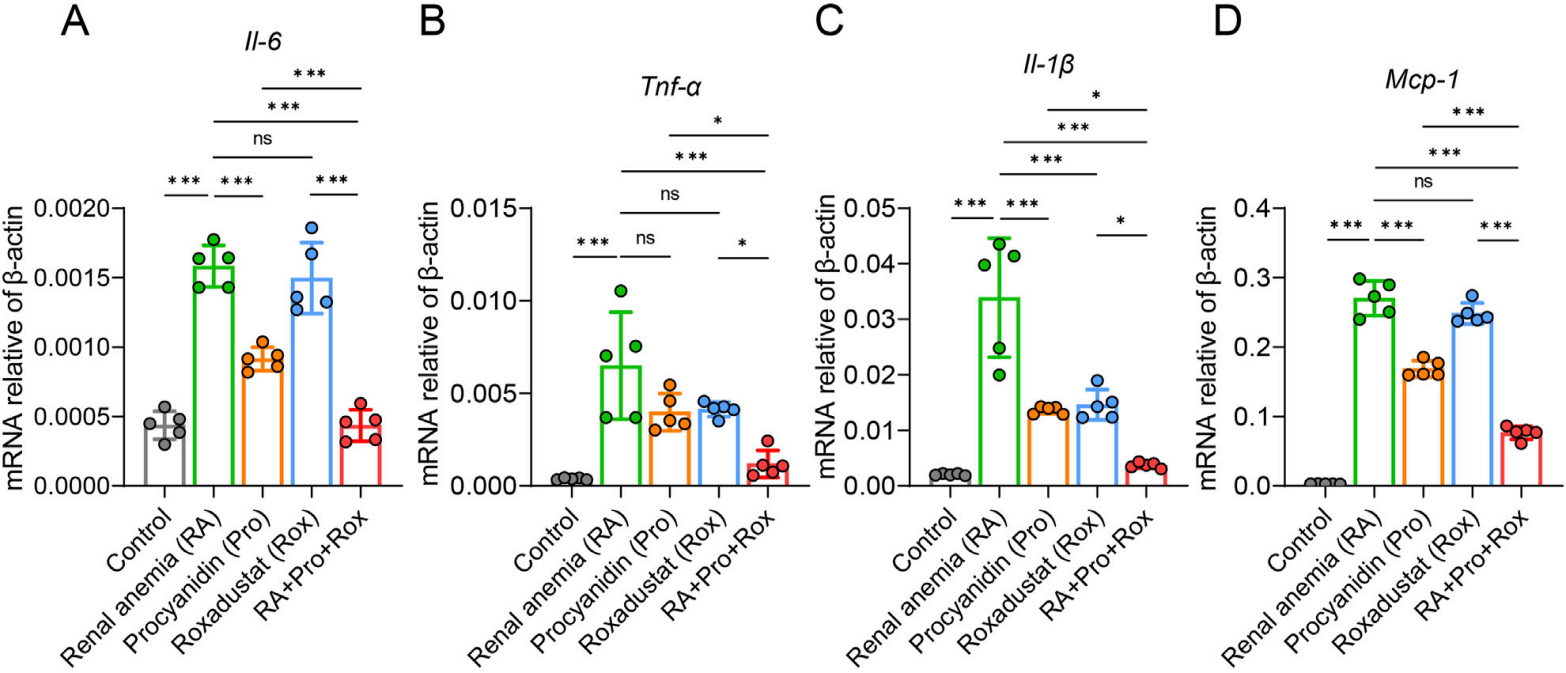
Procyanidin and roxadustat synergistically reduced kidney inflammation. Transcriptional expressions of inflammatory cytokines **(A)** *Il-6*, **(B)** *Tnf-α*, **(C)** *Il-1β* and **(D)** Monocyte chemotactic protein 1 (*Mcp-1*) in the kidney. Data are expressed as the mean ± standard deviation. One-way analysis of variance (ANOVA) and Tukey *post hoc* test were used to compare the group differences. Significant differences between groups were indicated by *, where * indicates *P* < 0.05; ** indicates *P* < 0.01; *** indicates *P* < 0.001; ns indicates no significant difference.

In mice, adenine can be transformed into 2,8-dihydroxyadenine, which deposits in the epithelial cells of renal tubules in a crystalline form, blocking renal tubules, inducing inflammation and ultimately leading to renal dysfunction. To examine the presence of inflammation in renal tissues, inflammatory cytokine expression levels across five groups were detected. Compared with control group, *Il-6, Tnf-α, Il-1β*, and *Mcp-1* expressions significantly elevated in the renal tissues of RA mice (Figures 5A–D). RA mice treated with procyanidin (Pro group), roxadustat (Rox group) or both (RA + Pro + Rox) exhibited varying degrees of decrease in inflammatory cytokine expression levels. The expressions of inflammatory factors in mice receiving both procyanidin and roxadustat were significantly lower than those receiving either procyanidin or roxadustat alone (Figures 5A–D).

### Procyanidin and roxadustat improved iron mobilization and iron absorption

**FIGURE 6:**
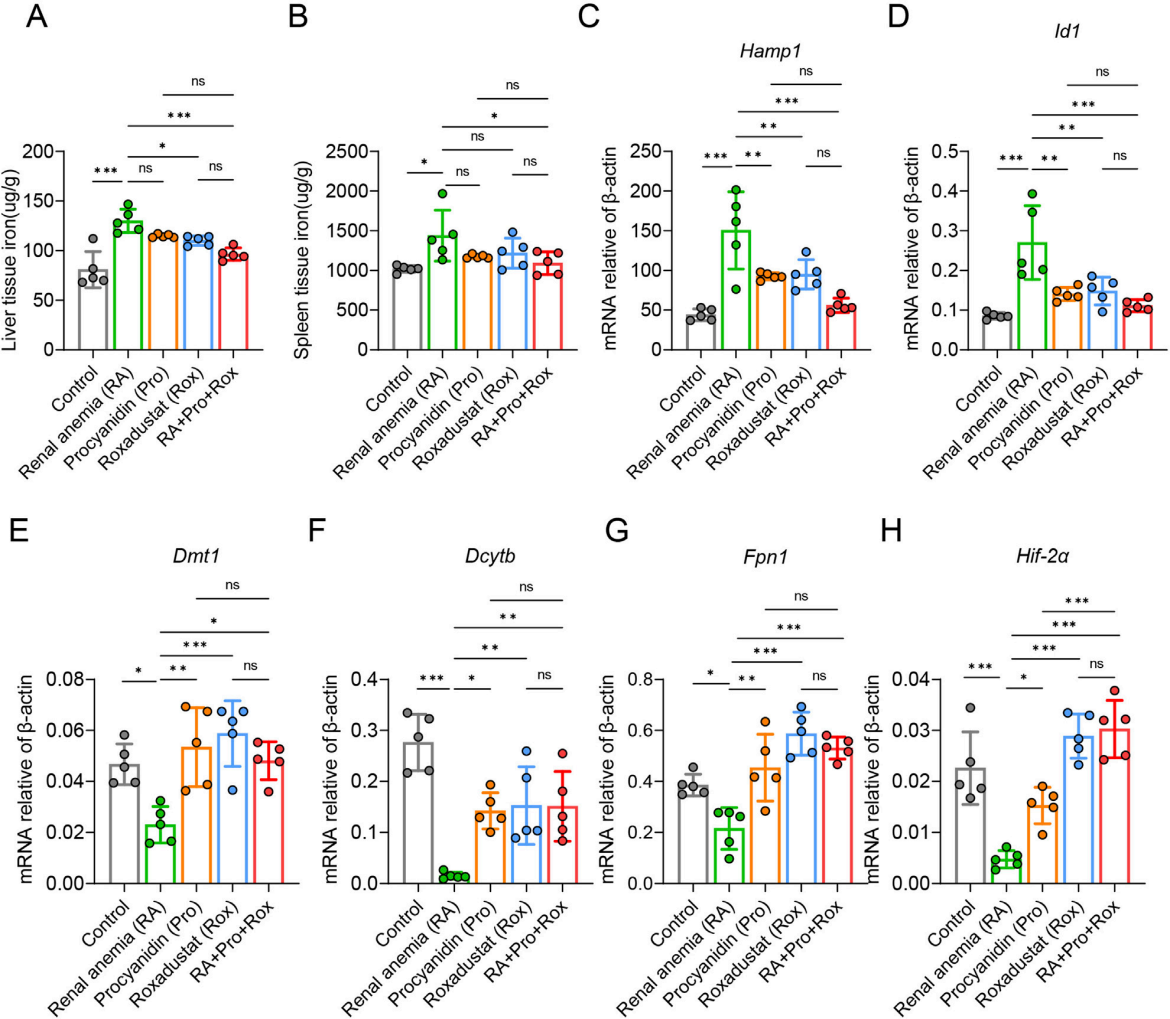
Procyanidin and roxadustat improved iron mobilization and iron absorption. **(A)** Liver and **(B)** spleen tissue non-heme iron concentrations. **(C)** Hepatic *Hamp1* and **(D)** *Id1* gene mRNA expressions. **(E)** *Fpn1*, **(F)** *Dmt1*, **(G)** *Dcytb*, and **(H)** *Hif-2a* gene mRNA expressions in small intestine. The data are expressed as the mean ± standard deviation. One-way analysis of variance (ANOVA) and Tukey *post hoc* test were used to compare the group differences. Significant differences between groups were indicated by *, where * indicates *P* < 0.05; ** indicates *P* < 0.01; *** indicates *P* < 0.001; ns indicates no significant difference.

Iron is a crucial precursor for hemoglobin synthesis in the body, and functional iron deficiency frequently coexists with CKD. To investigate iron metabolism in mice, hepatic and splenic tissue iron levels were measured across various groups. RA mice exhibited significantly elevated hepatic and splenic non-heme iron concentrations compared to the Control mice (Figures 6A,B). RA mice treated with roxadustat (Rox group) or both procyanidin and roxadustat (RA + Pro + Rox group) displayed reduced hepatic and splenic non-heme iron concentrations compared with RA mice. RA mice treated with procyanidin alone (Pro group) displayed unchanged non-heme iron concentrations. These findings suggest that roxadustat and the combined intervention effectively mobilized iron from iron storage organs.

Hepcidin (*Hamp1* gene), an essential hormone regulating iron metabolism, inversely correlated with tissue iron levels and is cleared in the kidney post-utilization. *Id1* is co-expressed with hepcidin upon iron can verify *Hamp1* expression. Compared with control mice, *Hamp1* and *Id1* expressions were elevated in RA mice (Figures 6C,D). RA mice treated with procyanidin (Pro group), roxadustat (Rox group) or both (RA + Pro + Rox group) displayed decreased *Hamp1* and *Id1* expressions when compared with untreated RA mice.

Next, we assessed the expressions of iron absorption genes, including *Dmt1, Dcytb, Fpn1*, and *Hif-2α* in the small intestine. RA mice exhibited reduced expression of these genes compared with control mice (Figures 6E–H). RA mice treated with procyanidin, roxadustat, or both, displayed upregulated *Dmt1, Dcytb, Fpn1* and *Hif-2α* in the small intestine.

## Discussion

In this study, we constructed a renal anemia model in mice using oral adenine administration, and used procyanidin and roxadustat to intervene the renal anemia mice separately and in combination. Our findings shows that procyanidin alone improved renal function and reduce the degree of renal injury, which can be reflected by improved hematological parameters, histopathological staining and expressions of genes related to renal inflammation and injury in mice. Roxadustat alone improved erythropoiesis via the induction of liver EPO. The combined intervention of procyanidin and roxadustat displayed synergistical effects on the improvement of renal function, renal injury, and erythropoiesis. These results demonstrated that the combination of procyanidin and roxadustat improved renal anemia by alleviating renal inflammation and injury, increasing HIF-2α and downstream EPO expression, and improving iron absorption and mobilization in the body.

The establishment of CKD animal models serves as a foundation for investigating renal anemia. There are several methods for the construction of animal models of CKD, such as 5/6 nephrectomy and unilateral ureteral obstruction (Bao et al., 2018). However, these two classical approaches are accompanied by several limitations, including but not limited to high mortality rates, and infrequent concurrent anemia symptoms in mice (Shobeiri et al., 2010). The adenine-induced CKD model possesses some merits. Adenine can be metabolized into 2,8-dihydroxyadenine, which forms crystals in proximal tubular epithelial cells, blocking the tubules and subsequently eliciting inflammation, fibrosis, and eventually induces renal anemia (Yokozawa et al., 1985; Yokozawa et al., 1984; Yamato et al., 2020). These pathological changes of kidney in adenine-induced model allow us to evaluate whether procyanidin or/and roxadustat directly improves kidney function.

Based on previous studies, intraperitoneal injection of 10 mg/kg roxadustat has certain nephroprotective effect, because it reduces serum creatinine and urea nitrogen in folic acid-induced acute kidney injury models (Li et al., 2021). However, in the present study, oral gavage of 5 mg/kg roxadustat appeared to have no obvious effect on kidney function in adenine-induced renal injury, though it still effectively improved erythropoiesis. In mice receiving 5 mg/kg roxadustat, no change was found in kidney wight, glomerular injury score, tubular injury score, creatinine levels, urea levels and gene marker reflecting kidney injury (Figure 3), although roxadustat was shown to reduce renal fibrosis (Figure 4). The reduction of fibrosis could be attributed to the reason that roxadustat might attenuate signaling pathways involved in fibrosis, such as TGF-β pathway (Zheng et al., 2023). The major efficacy of roxadustat was erythropoiesis promotion via inhibiting prolyl hydroxylase (PHD), stabilizing HIFs, and upregulating EPO expression. HIFs are multifunctional, and therefore HIF activation in patients with renal anemia may also have effects other than erythropoiesis (Haase, 2021). For example, the stabilized HIF-2α by roxadustat also promoted the expression of cellular iron exporter ferroportin (Fpn) (Figure 6G), and mobilized iron export out of renal cells, which may also contribute to the reduction of iron-induced renal fibrosis (Patino et al., 2023; Wu et al., 2024).

Procyanidin possesses antioxidative, anti-inflammatory, and renal protective effects, and no adverse effects have been found so far in animals after ingesting high doses of procyandin (Woo et al., 2011; Lai et al., 2018; He et al., 2018). In Sprague-Dawley rats received kidney ischemia reperfusion, orally pre-feeding with 50 mg/kg/d of procyanidin for 2 weeks reduced the oxidative stress in renal tissues, and improved renal injury caused by renal ischemia reperfusion (Ashtiyani et al., 2013). Research reports have indicated that procyanidin can stimulate lymphocyte transformation, enhance lysosomal enzyme activity, and boost the phagocytic capacity of macrophages (Tong et al., 2011). Furthermore, procyanidin reduces the airway inflammation in mice through decreasing inflammatory cell infiltration, reducing the expression of Th2 cytokines, and lowering serum IgE levels (Lee et al., 2012). Procyanidin reduced the loss of chondrocytes and proteoglycans via its anti-inflammatory effects in a rat model of osteoarthritis (Woo et al., 2011). A previous study also showed that oral administering 200 mg/kg/d of procyanidin improved renal function decline caused by acute renal injury and reduce oxidative levels in renal tubular cells in mice (Liu et al., 2020). In this study, we used a comparable dosage of procyanidin (250 mg/kg/d) to treat mice with renal anemia, which expands the disease spectrum for procyanidin.

Prolonged or high doses of roxadustat may lead to a significant rise in EPO, which not only promotes erythropoiesis but also thrombopoiesis, resulting in coagulation (Chen et al., 2019). It may involve two mechanisms. First, reactive thrombocytosis from iron deficiency secondary to enhanced erythroid demand: roxadustat-stimulated erythropoiesis increases iron requirements, and unmet demand can trigger compensatory platelet elevation, consistent with clinical links between iron deficiency and reactive thrombocytosis (Sharma et al., 2023). Though roxadustat improved iron mobilization in our study, residual functional iron imbalance may have favored this pathway. Second, shared progenitor cell activation: erythrocytes and platelets derive from common myeloid progenitors (CMPs) and megakaryocyte-erythroid progenitors (MEPs); roxadustat-induced HIF activation not only promotes erythropoiesis via EPO but may also enhance megakaryopoiesis by regulating HIF-responsive genes in MEPs (Wolff and Humeniuk, 2013). Given the antioxidant and kidney-protective properties of procyanidin, we combined it with roxadustat to mitigate the side effects of roxadustat. Unexpectedly, roxadustat with procyanidin displayed a synergistical effects on erythropoiesis, kidney injury, kidney function, and iron metabolism (Figures 2A, 3, 6). Our results showed that 5 mg/kg roxadustat already significantly elevated platelet indices. Procyanidin was reported to have antithrombotic effect (Marcińczyk et al., 2021). The combination of roxadustat with procyanidin significantly reverted the elevated platelet indices induced by roxadustat (Figures 2G,H). The concentrations of roxadustat and procyanidin we used may not be optimal, and considering the 3R principle, we limited the number of animals in each group. Further studies are still needed. Future research may focus on whether different dosage combinations of roxadustat and procyanidin yield better outcomes, and continue to evaluate the clinical applicability and efficacy of this combination. It is hoped that this combination will be transformed clinically and better treat renal anemia.

In conclusion, this study demonstrates that procyanidin and roxadustat synergistically ameliorate the mouse model of renal anemia. Our findings suggest that procyanidin may reduce the need of drug doses in renal anemia treatment and alleviate related adverse effects.

## Data Availability

The raw data supporting the conclusions of this article will be made available by the authors, without undue reservation.
