# Supplementary figures and images for: Procyanidin displayed a synergistic effect with roxadustat on renal anemia in mice
Source: Front Pharmacol. 2025 Nov 12;16:1678846. doi: 10.3389/fphar.2025.1678846 (PMC12646935; doi:10.3389/fphar.2025.1678846)

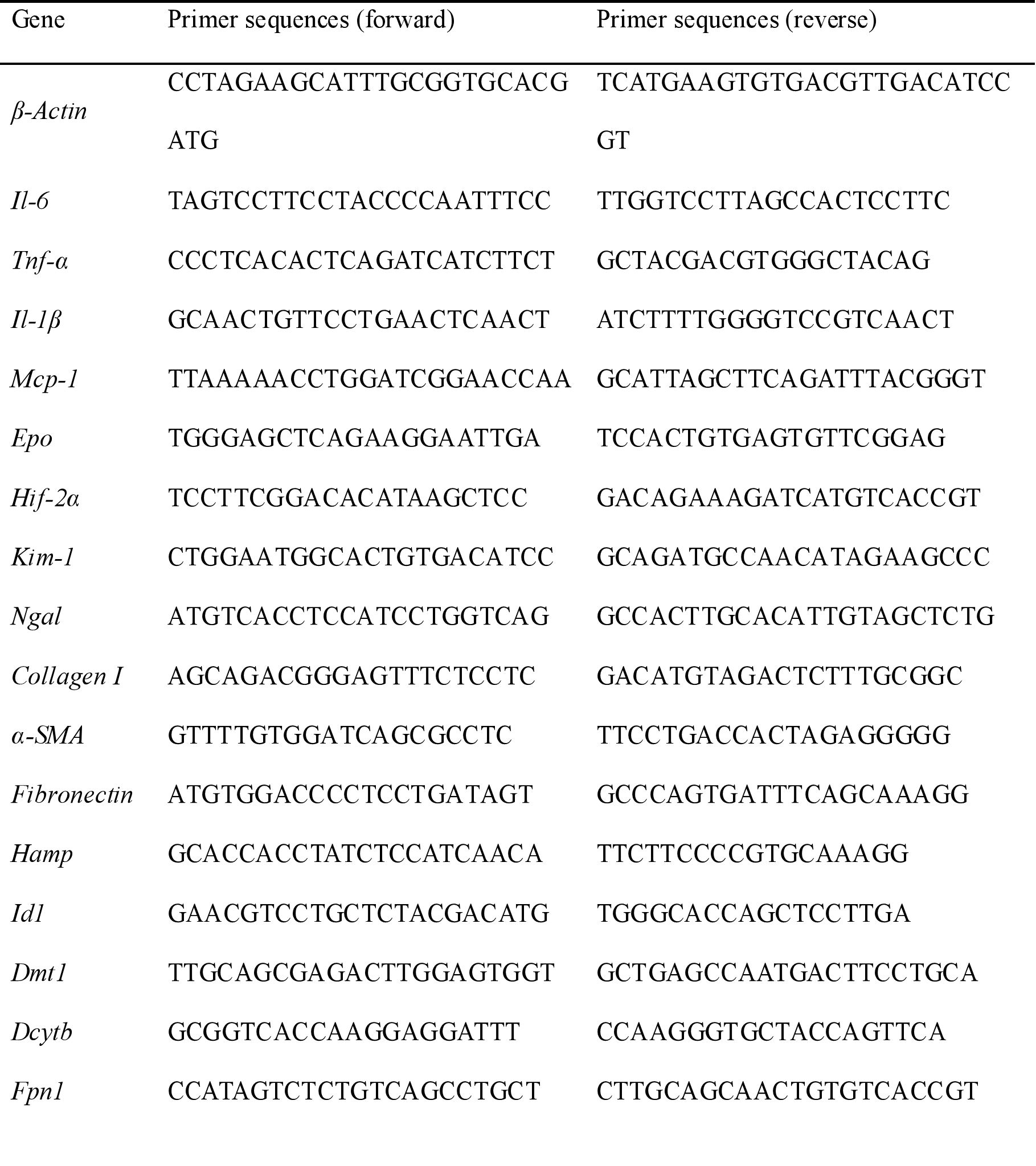

Supplement: Supplementary file 1 [file Table1.docx]
